# The Relationship Between Access to Abortion and Mental Health in Women of Childbearing Age: Analyses of Data From the Global Burden of Disease Studies

**DOI:** 10.7759/cureus.31433

**Published:** 2022-11-13

**Authors:** Ravi P Rajkumar

**Affiliations:** 1 Psychiatry, Jawaharlal Institute of Postgraduate Medical Education and Research, Pondicherry, IND

**Keywords:** intimate partner violence, mental health, reproductive rights, depression, anxiety, medical termination of pregnancy, abortion

## Abstract

Background

It has often been suggested that restricting access to legal abortion could have a negative impact on the mental health of women seeking this procedure. The recent judgment of the United States Supreme Court in the Dobbs case has brought the question of the psychological impact of changes in abortion policy into focus. The results of studies on the link between induced abortion and mental health are contradictory, and interpreting the results of these studies is complex due to a multitude of potential confounding factors. However, there is little data on the relationship between the availability of legal abortion and its effects on the mental health of women of childbearing age in the general population.

Objective

The objective of the current study was to examine cross-sectional and longitudinal associations between access to legal abortion and the prevalence of common mental disorders in women of childbearing age at a cross-national level while correcting for potential confounding factors.

Methods

The current study employs both cross-sectional and longitudinal analyses of nation-level data for 197 countries and regions. As data on global abortion policy were compiled in 2009 and 2017 by the Guttmacher Institute, data on access to legal abortion for these years were examined in relation to the prevalence of anxiety disorders and depression in women of childbearing age for each country, obtained from the Global Burden of Disease Studies for the most recent subsequent year (2010 and 2019). The relationship between changes in abortion policy and changes in the prevalence of these disorders in each country was examined for the aforementioned period. All analyses were adjusted for the potential confounding effects of gender development, gender inequality, and intimate partner violence. Cross-sectional associations were examined using Pearson's bivariate and partial correlations, while longitudinal associations were examined using a general linear model and analyses of covariance.

Results

At the cross-sectional level, broad legal access to abortion was associated with a lower prevalence of depression in women aged 25-49 years, however, this association was not significant after correcting for measures of gender development, gender inequality, and intimate partner violence. At the longitudinal level, a slight but significant decrease in the prevalence of anxiety disorders in women aged 25-49 years was observed in countries where access to legal abortion was broadened in the period 2009-2017. This association remained marginally significant after adjustment for the above confounders.

Conclusions

The current study suggests that there is a modest relationship between access to legal abortion and its effects on the mental health of women aged 25-49 years. However, this relationship appears to be largely indirect in nature and influenced by factors, such as gender development, gender inequality, and intimate partner violence. These results may lead to further exploration of the links between reproductive rights and mental health of women in the general population and draw attention to the influence of gender inequality and intimate partner violence on mental health of women of childbearing age.

## Introduction

On June 24, 2022, the Supreme Court of the United States of America issued its judgment on the case Dobbs vs. Jackson Women’s Health Organization, reversing the decision issued in the case Roe vs. Wade (1973) which guaranteed legal access to abortion at the federal level [1]. The consequences of this decision extend far beyond the boundaries of abortion access, both in the United States and at the international level [2-4]. As a result, broader questions on the individual and societal consequences of changes in the legality of abortion have come into focus. While some of this debate is of an ethical or philosophical nature [5], much of it concerns the physical, psychological, and social consequences of such changes, particularly when they are in the direction of reduced access [6,7]. These considerations should be viewed against the background of global statistics on abortion and abortion access. An analysis of worldwide data estimated that there were over 120 million unplanned pregnancies in the period 2015-2019 and that over 60% of these pregnancies ended in abortion. Moreover, it was observed that women sought access to safe and legal methods of termination of pregnancy even in countries or regions where significant restrictions were in place, leading to the paradoxical finding that a greater proportion of unplanned pregnancies ended in abortion in such regions [8]. In contrast with the situation in the United States, the global trend during this period has been towards greater access to legal abortion - it is estimated that in the period 2000-2018, 27 countries enacted legislation that would reduce some of the barriers faced by women seeking abortion [9]. More recently, abortion access has also been broadened in Ireland and Argentina [10,11].

The effects of abortion on the mental health of women have been studied extensively over the past five decades, however, even when published evidence is available, its interpretation is often colored by ideological pre-suppositions [12]. Several research studies have presented evidence of a link between abortion and adverse mental health outcomes, but such a link may not be significant after adjusting for individual and social factors that themselves affect mental health. Examples of these factors are current relationship status and stability, intimate partner violence, social support, socioeconomic status, concurrent substance abuse, childhood attachment, and a history of abuse or neglect in childhood [13,14]. Moreover, the relationship between mental health, unplanned pregnancy, and abortion is not unidirectional. The presence of common mental disorders is itself associated with an increased rate of unplanned pregnancy in young women [15,16], and several factors that can themselves increase the risk of depression, such as childhood abuse, economic difficulties, gender-based violence, and relational problems with a partner or spouse, appear to influence the link between abortion and subsequent mental health [17]. In a critical review of the existing literature, Reardon concluded that though there was consistent evidence of a link between abortion and adverse mental health outcomes, this relationship was confounded by factors such as the experience of abortion itself and pre-existing risk factors, leading to significant difficulties in establishing causality [18]. Social and cultural factors may also play a significant role in influencing these outcomes - longitudinal studies from the Netherlands [13], Finland [19,20], and South Africa [21] did not demonstrate significant elevations in psychological morbidity following abortion, in contrast to studies from New Zealand [17], Norway [22], and the United States [23,24].

Though there is a significant body of literature examining the mental health effects of abortion, there are fewer studies examining the mental health consequences of abortion refusal, or of abortion restrictions in general. A study of over 800 women seeking abortion in the United States documented elevated levels of regret and anger following denial of abortion, but participants in this study were followed up only for one week [25]. Subsequently, a prospective longitudinal study of the United States' women, the Turnaway Study, identified a short-term (one week) increase in anxiety in women denied an abortion, however, this effect was not sustained when participants were followed up over a period of five years, and overall mental health outcomes were comparable with those women who underwent abortion [26]. A subsequent qualitative study, based on a sub-group of 161 women from the Turnaway Study, found that negative emotions gradually decreased and positive emotions increased during the course of pregnancy and after childbirth in these women. These outcomes were influenced by factors such as prior beliefs regarding abortion, social support, postpartum mother-infant bonding, and positive life events [27]. Despite the paucity of empirical evidence, it is often suggested that abortion restrictions could contribute significantly to mental morbidity in women of childbearing age, particularly in those with other risk factors for the development of depression [28,29].

A limitation of much of this literature is its focus on the unique situation observed in the United States, where abortion is an emotionally and politically charged issue [2,30]. The availability of global or cross-national data on the links between abortion and mental health, even if provisional in nature, would provide a broader context for both this literature and the discourse surrounding it, and would be of use to both the general public and to experts, particularly in settings whose socioeconomic realities differ widely from those of the United States or Western Europe [31-33]. Moreover, abortion access is intimately connected to broader issues of women’s rights, autonomy, and empowerment at both the individual and societal levels [34,35]. Therefore, any changes in this parameter are likely to affect mental health not only in women seeking abortion, but in women of childbearing age and, perhaps to a lesser extent, women in the general population, in the manner of a “ripple” or “halo” effect [36-38].

The aim of the current study is to examine cross-sectional and longitudinal associations between access to legal abortion and mental health of women of childbearing age, over the period 2010-2019, while adjusting for general measures of gender equality and inequality. Owing to the contradictory nature of the findings in the existing literature, described above, no priori hypothesis on the nature of these associations can be formulated, and this study is exploratory in nature.

## Materials and methods

Study design

**Figure 1:**
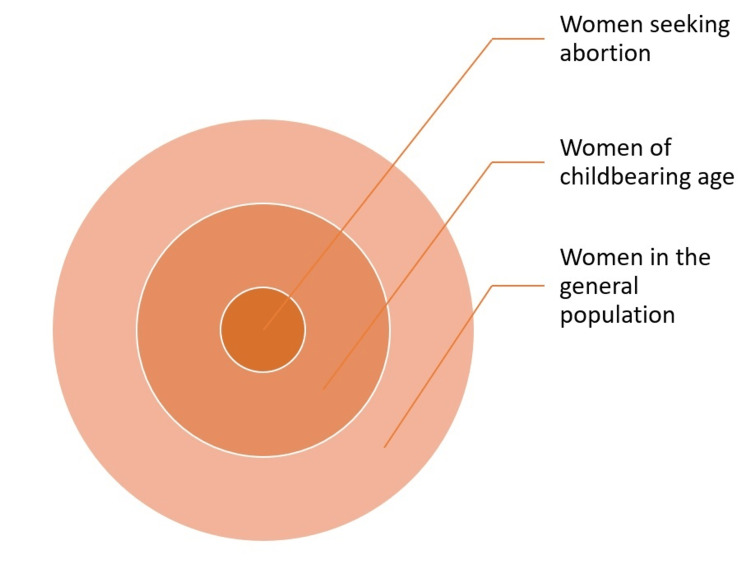
Wider effects of access to abortion on the mental health of women. This figure depicts the potential mental health impacts of abortion legislation on women. Though the largest impact would be expected in women seeking an abortion for various indications, it is possible that significant (though smaller) impacts may be observed in women of childbearing age and even in women in the general population who do not belong to these two groups.

The current study is a cross-national, ecological analysis of the correlations between access to legal abortion and its effects on the mental health of women of childbearing age. It involves both cross-sectional and longitudinal analyses of the relationship between access to legal abortion and the prevalence of common mental disorders - depression and anxiety disorders - in women belonging to this age category for each country. The rationale for this analysis has been discussed above and is depicted graphically in Figure 1.

Study period

This study covered the time period 2009-2019. In the cross-sectional analysis, evidence of a positive or negative relationship between different grades of access to legal abortion and the prevalence of these disorders was examined for the periods 2009-2010 and 2017-2019. In the longitudinal analyses, the relationship between changes in abortion access over the period 2009-2017 was examined in relation to changes in the prevalence of these disorders in the period 2010-2019. In order to ensure that any associations identified were not due to the effect of potential confounding variables, all analyses were adjusted for these variables.

Sample size and eligibility criteria

As the current study involved cross-national analyses, sample size was not calculated a priori but was based on the availability of relevant data. Data on abortion access were available for 197 countries and regions, but only 194 of these countries were represented in the Global Burden of Disease studies. The three countries for which mental health data were not available were excluded. Hence, the final sample size for this study consisted of 194 countries.

Study variables and their sources

Abortion

Data on legal access to abortion were obtained from two reports published by the Guttmacher Institute in the years 2009 and 2017 [39,40]. The Guttmacher Institute is a non-governmental organization (NGO) based in the United States and is “committed to advancing sexual and reproductive health and rights worldwide” through research, advocacy, and communication [41]. The 2009 and 2017 reports provide information on legal access to abortion in 197 countries and regions worldwide, based on information supplied by each country. In these reports, access to abortion is divided into the following six categories, arranged from most to least restrictive: 1 - prohibited, 2 - permitted only to save the life of the mother, 3 - permitted to save the life of the mother or preserve her physical health, 4 - permitted to save the life of the mother or preserve her physical or mental health, 5 - all of the above, and also permitted on socioeconomic grounds, and 6 - no restriction. An alternative, dichotomous classification is also provided in both publications, in which categories 1-4 are grouped together as “highly legally restricted” and categories 5-6 are grouped as “broadly legal.” As the current study is exploratory in nature, data on legal abortion in each country were entered using both classifications, and data analyses were carried out separately for each. Information on changes in abortion legislation in the period 2009-2017 was also entered for each country, based on a comparison of the two reports, under the following three categories: “no change,” “broader access,” and “restricted access.”

Mental Health

Information on mental health was obtained from the Global Burden of Disease (GBD) Study database [42]. The Global Burden of Disease studies provide comprehensive and up-to-date estimates of the prevalence of common mental disorders - anxiety disorders and depression - both for each country as a whole, and according to gender and age groups, based on the most recent data available from each country as well as statistical models [43]. As the focus of this study was on women of childbearing age, data on the prevalence of depression and anxiety disorders were retrieved for the age groups 10-24 and 25-49 for each country for the years 2010 and 2019. Age groupings were provided by the database and cannot be modified by the user; as it is likely that the mental health impact of changes in abortion policy would affect younger and older women differently, these two groups were selected as being the most likely to yield findings relevant to the current research question. To examine changes in these variables over time, the increase or decrease in the prevalence of each disorder in the period 2010-2019, defined as the percentage of change from the baseline (2010) value, was calculated for both age groups for each country.

Confounding Factors

The issue of legal access to abortion does not exist in a social vacuum - it reflects broader social, cultural, and political realities about women’s rights and empowerment in a given country [34-38]. Therefore, even if an association between the availability of abortion and women’s mental health can be demonstrated, it is important to ensure that such an association is not due to the confounding effects of more general measures of gender equality or inequality. In order to control for such effects, both cross-sectional and longitudinal analyses were corrected for two potential confounders. The first, the Gender Inequality Index (GII), is a composite measure of gender inequality based on measures of reproductive health, empowerment, and labor force participation. This measure is scored from 0 (no inequality) to 1 (highest levels of inequality). The second, the Gender Development Index (GDI), measures gender development as a composite of the following three basic indicators: life expectancy, education, and income. The GDI is scored as the ratio of the composite score on these indicators for men and women. Information on these variables was obtained from the United Nations’ Human Development Reports [44,45]. Data on the GII were available for both 2010 and 2019 and were included in both the cross-sectional and longitudinal analyses; data on the GDI were available only for 2019 and were used only in the cross-sectional analysis.

In addition to these general measures of social and economic development, several studies have identified intimate partner violence (IPV) as being one of the key factors involved in a woman’s decision to seek an abortion, both in developed and developing countries [46-48]. Intimate partner violence is itself a significant risk factor for the development of depression in women [49]. In order to correct for the possible confounding effect of this form of trauma, data on the estimated lifetime prevalence of IPV in women of childbearing age in each country were retrieved from a 2018 World Health Organization Report on this subject [50]. As this data were available only for the year 2018, it was used only in the cross-sectional analyses.

Data analyses

All statistical analyses were carried out using the Statistical Package for Social Sciences (SPSS), version 28.0 (Armonk, NY: IBM Corp.). In the cross-sectional analyses, estimated differences in the estimated national prevalence of anxiety disorders and depression for women in the age groups 10-24 years and 25-49 years were examined according to the level of access to legal abortion in each country, using both classificatory schemes outlined in the Guttmacher Institute’s reports. A one-way analysis of variance (ANOVA), with Tukey’s honestly significant difference (HSD) post-hoc test, was used to assess significant differences in the prevalence of these disorders across the six different levels of abortion access, while the independent samples ­­t­-test was used when countries were classified dichotomously as “highly restricted” and “broadly legal.” As a confirmatory analysis, Spearman’s rank correlation coefficient (ρ) was also computed to test for significant correlations between access to abortion (as an ordinal variable ranging from 1 to 6) and the prevalence of anxiety disorder and depression. Analyses were carried out separately for the years 2010 and 2019.

Bivariate (Pearson’s r) correlations were also examined for all three confounding variables of interest, namely the GII (2010 and 2019), GDI (2019), and the estimated prevalence of IPV (2019). Based on the results of these correlations, analyses of covariance (ANCOVA) were carried out using those confounders identified as significant on bivariate analyses, to examine if any observed association between abortion access and women’s mental health remained significant after adjusting for these confounders.

In the longitudinal analyses, the percentage of change in the prevalence of anxiety disorders and depression for both age groups was computed for each country. Differences in this percentage were compared across countries based on whether there was no change, broader access, or restricted access to legal abortion in the period 2009-2017, using the independent samples t-test. To confirm any results obtained using this method, a general linear model was also computed to examine the effects of time and time×group on changes in the prevalence of these disorders. Finally, an analysis of covariance (ANCOVA) was carried out to examine whether any observed associations remained significant after correcting for the GII, GDI, and the prevalence of IPV. All statistical tests were two-tailed, and statistical significance was set at p<0.05.

Ethical approval

As this study was based on anonymous, country-level data and did not involve individual human subjects, there was no requirement for informed consent or approval as per the relevant institutional and national guidelines.

## Results

Legal access to abortion, 2009-2017

Information on access to legal abortion was available for a total of 197 countries and regions. Abortion access in 2009 was classified as “restricted” in 127 countries (64.5%) and “broad” in 70 countries (35.5%); in 2017, the respective frequencies were 124 (62.9%) and 73 (37.1%). Relative frequencies of the different levels of abortion access for each year, using the six categories outlined by the Guttmacher Institute publications, are presented in Table 1.

**Table 1:** Access to legal abortion in 197 countries and regions, 2009-2017. The table is adapted from Singh et al. (2009) [39] and Singh et al. (2017) [40].

Level of access	2009	2017
Prohibited	32 (16.2%)	26 (13.2%)
To save the mother’s life	36 (18.3%)	38 (19.3%)
To save the mother’s life or preserve physical health	36 (18.3%)	36 (18.3%)
To save the mother’s life or preserve physical or mental health	23 (11.7%)	24 (12.2%)
Life-saving, health, and socioeconomic indications	14 (7.1%)	13 (6.6%)
No restrictions	56 (28.4%)	60 (30.5%)

Cross-sectional associations between abortion access and mental health

Results of the associations between the different categories of legal access to abortion and the prevalence of anxiety disorders and depression in women aged 10-24 years and 25-49 years are presented in Table 2 for the six-category classification and Table 3 for the dichotomous classification. It was found that no significant difference in the prevalence of these disorders for either age group was found when the six-category classification was used. Although there was a trend towards a lower prevalence of depression in women aged 25-49 years in countries with less restrictions on abortion (F=1.99, p=0.081), no significant difference between groups was observed on post-hoc testing. On the other hand, when a dichotomous classification (restricted vs. broad access) was used, it was observed that the prevalence of depression in women aged 25-49 years was significantly lower in countries with broad access in both 2010 (t=2.39, p=0.018) and 2019 (t=2.87, p=0.005). Similar results were obtained when Spearman’s correlation coefficients were computed for the six-category measure - broader access to abortion was negatively correlated with the prevalence of depression in women aged 25-49 years both in 2010 (ρ= -0.17, p=0.017) and in 2019 (ρ= -0.17, p=0.021), though the magnitude of this correlation was modest. A trend towards a negative association was also observed between broader access to abortion and the prevalence of anxiety disorders in women aged 25-49 years, but only in the year 2019 (ρ= -0.13, p=0.064).

**Table 2:** Associations between legal access to abortion (six categories) and the prevalence of common mental disorders in women aged 10-24 years and 15-49 years. "F" denotes analysis of variance (ANOVA) test statistic and "p" denotes statistical significance level (p-value).

Year	Depression (%), women 10-24 years	Anxiety disorders (%), women 10-24 years	Depression (%), women 25-49 years	Anxiety disorders (%), women 25-49 years
2009	F=0.37, p=0.870	F=0.53, p=0.752	F=1.36, p=0.242	F=0.98, p=0.432
2017	F=0.08, p=0.996	F=0.79, p=0.555	F=1.99, p=0.081	F=1.03, p=0.402

**Table 3:** Associations between legal access to abortion (dichotomous) and the prevalence of common mental disorders in women aged 10-24 years and 15-49 years. *P-value significant at <0.05. **P-value significant at <0.01. "t" denotes independent samples t-test statistic and "p" denotes statistical significance level (p-value).

Year	Depression (%), women 10-24 years	Anxiety disorders (%), women 10-24 years	Depression (%), women 25-49 years	Anxiety disorders (%), women 25-49 years
2009	t=0.16, p=0.872	t= -0.41, p=0.661	t=2.39, p=0.018*	t=0.95, p=0.341
2017	t=0.50, p=0.618	t= -0.67, p=0.501	t=2.87, p=0.005**	t=0.60, p=0.547

Effect of confounding variables on cross-sectional associations

Among the three confounding variables examined in this study, data were available for the year 2010 only for the GII. This variable showed a modest negative correlation with the prevalence of anxiety disorders, but only in women aged 10-24 years (r= -0.19, p=0.024). For the year 2019, data on all three confounding variables were available. The GII was again negatively correlated with the prevalence of anxiety disorders in women aged 10-24 years (r= -0.39, p<0.001) and 25-49 years (r= -0.27, p<0.001), but was positively correlated with the prevalence of depression in women aged 25-49 years (r=0.32, p<0.001). The GDI was negatively correlated with depression only in women aged 25-49 years (r= -0.40, p<0.001), while the prevalence of IPV was positively correlated with depression in this age group (r=0.28, p<0.001).

Based on these analyses, an analysis of covariance for the year 2019 was carried out using the GII, GDI, and IPV as covariates, the prevalence of depression in women aged 25-49 years as the dependent variable, and abortion access (dichotomous) as the fixed factor. In this model, the association between abortion access and depression in women aged 25-49 years was no longer significant (F=0.531, p=0.468), as was the association between the GII and depression (F=0.221, p=0.639). However, the associations between the GDI and depression (F=8.212, p=0.005) and the prevalence of IPV and depression (F=24.339, p<0.001) remained statistically significant.

Longitudinal analyses

Prior to longitudinal analyses, paired samples t-tests were carried out to test for the possibility of secular trends in the prevalence of anxiety and depression in women of both age groups over the period 2010-2019. The results of these tests revealed a slight but significant increase in the prevalence of anxiety disorders in women aged 25-49 years over this period (6.52% in 2019 vs. 6.47% in 2010; t=2.06, p=0.04), but no significant differences for depression in either age group.

Comparisons of the changes in the prevalence of these disorders between countries with no change in legal access to abortion and those with a liberalization of legal indications over the period 2009-2017 are presented in Table 4. In this analysis, it was observed that in countries in which access to abortion was broadened in the period 2009-2017, there was a slight decrease in the prevalence of anxiety disorders in women aged 25-49 years (-1.26%), as opposed to a slight increase in countries with no policy change (+1.19%). This difference was statistically significant (t=2.31, p=0.022). No difference was observed for depression or for women aged 10-24 years. This finding was partly confirmed through a general linear model which showed a trend towards a time×group interaction for this variable (F=3.64, p=0.058) as well as a significant effect for time (F=5.44, p=0.021).

**Table 4:** Associations between changes in abortion access and changes in the prevalence of common mental disorders in women. *P-value significant at <0.05. Values for the percentage of change are given as mean (standard deviation).

Disorder and age group	Change in prevalence (%) in countries with no change in abortion access	Change in prevalence (%) in countries with liberalization of abortion access	T-test statistic (p)
Depression, women aged 10-24 years	0.45 (6.65)	3.52 (4.70)	-1.63 (0.105)
Anxiety disorders, women aged 10-24 years	0.99 (4.07)	0.47 (3.31)	0.45 (0.652)
Depression, women aged 25-49 years	0.71 (4.75)	0.43 (5.61)	0.21 (0.835)
Anxiety disorders, women aged 25-49 years	1.19 (3.56)	-1.26 (5.34)	2.31 (0.022)*

Effect of confounding variables on longitudinal associations

When examining correlations between the three confounders of interest and changes in the prevalence of anxiety disorders and depression over the period 2010-2019, it was found that the GII was positively correlated with increases in the prevalence of anxiety disorders in women aged 10-24 years (r=0.34, p<0.001) and 25-49 years (r=0.16, p=0.044), while the prevalence of IPV was positively correlated with increases in the prevalence of anxiety disorders in the 10-24 years age group (r=0.20, p=0.016). For the GII, longitudinal data (for the years 2010 and 2019) were available. When examining correlations between changes in GII and mental health outcomes, it was found that the percentage of change in the GII was negatively correlated with changes in the prevalence of anxiety disorders in the age group 10-24 years (r= -0.33, p<0.001).

On the basis of these results, an analysis of covariance was carried out using the GII (for year 2019) as a covariate, the percentage change in the prevalence of anxiety disorders in women aged 25-49 years as the dependent variable, and changes in abortion policy as the fixed factor. In this model, a trend remained towards a significant association between changes in abortion policy and changes in the prevalence of anxiety disorders in this age group even after adjusting for the GII (F=3.91, p=0.05), while the association with the GII remained significant (F=4.12, p=0.044).

Relationships between abortion access and confounding variables

As a final set of analyses, the relationship between abortion access and the three confounders examined in this study - the GDI, GII, and the prevalence of IPV - were also examined. The GII was positively associated with greater restrictions on abortion access in both 2010 and 2019 when abortion access was classified according to six categories (F=2.30, p=0.049 in 2010; F=23.13, p<0.001), however, when classified dichotomously, there was a significant association for 2019 (t=10.63, p<0.001) but not for 2010 (t=1.43, p=0.157). The estimated prevalence of IPV (F=10.37, p<0.001) and the GDI (F=6.820, p<0.001) for 2019 were both inversely associated with access to legal abortion; greater restrictions were associated with a higher prevalence of IPV and a lower GDI.

## Discussion

Research on the relationship between abortion and mental health has often been the source of controversy. Much of this controversy arises from differences in cultural and religious beliefs about this procedure, leading to differences in the way harm is conceptualized [51,52]. It is not the aim of the current study to arrive at moral or ethical conclusions about the “rightness” or “wrongness” of abortion, but to expand the scientific evidence base in this field. More specifically, this study was carried out to assess whether access to abortion has a broader effect on women of childbearing age, over and above its immediate effects on women seeking an abortion for various indications (Figure 1). This question is of importance when considering the possible societal impact of changes in abortion legislation, whether these are in the direction of greater restriction [1-4] or of broader access [10-11].

With regards to the cross-sectional analyses, it was observed that the prevalence of depression was lower in countries with broader access to legal abortion, but only in women aged 25-49 years. This result was consistent across both time points included in this study. Taken at face value, such a finding would appear to suggest that less restrictive abortion policies are negatively correlated with depression, though this effect is mediated by age. As these analyses are cross-sectional, it is not possible to arrive at conclusions regarding causality, however, it appears more likely to assume that the legal provisions might lead to reduced depression than to assume the reverse. This is because mental health indications, including depression, are themselves associated with increased rates of unplanned pregnancy and requests for induced abortion; if levels of depression among women of childbearing age were driving a liberalization in abortion policy, one would expect findings opposite to those of the current study [15,16,53,54]. The possible mechanisms that could mediate the association in this study include (a) positive effects of abortion on depression in those seeking this procedure for mental health indications, (b) positive effects of abortion on mental health in women living in situations characterized by chronic stress or trauma, such as IPV, and (c) a more general effect among women of childbearing age, in which knowing that legal abortion is available as an option in the event of unforeseen medical, psychological, or interpersonal problems gives them a greater sense of autonomy and control over their reproductive choices [55-57]. While these explanations are plausible, it is also significant that after adjusting for confounding factors, this relationship was not significant; instead, the prevalence of depression in women in this age group was associated with lower levels of gender development and higher rates of intimate partner violence. Therefore, the most likely conclusion is that the link between abortion access and depression in older women of childbearing age is indirect, and is more directly related to women’s rights and social status in both the personal and political spheres [58,59]. This is supported by the additional analyses presented in this study, which show that abortion access is strongly and negatively associated with measures of gender inequality and intimate partner violence, and positively associated with a measure of gender development.

The results of the longitudinal analyses provide a different perspective. In these analyses, countries in which indications for legal abortion were broadened in the period 2009-2017 were found to have a slight but significant decrease in the prevalence of anxiety disorders in women aged 25-49 years, as opposed to a slight increase in countries with no policy change. This difference was statistically significant and remained largely unchanged after adjustment for potential confounders. This finding should be interpreted with caution, as it was based on changes in national abortion policy in only 14 countries. However, it does suggest that broader access to legal abortion may have a modest, positive effect on some aspects of mental health in women of childbearing age. As there were no instances of a restriction in abortion policy in any country in the current dataset, the converse - that more restrictive abortion policies may adversely impact the mental health of some women in the general population - cannot be affirmed based on the current results.

The current study, though preliminary in nature, adds a further dimension to the ongoing debate on the relationship between termination of pregnancy and mental health. Most of the literature has focused on the short- and long-term impact of this procedure and its attendant circumstances on those women undergoing it [60]. The results presented above suggest that when considering the mental health consequences of abortion policies, it is important to think beyond impacts on actual users of abortion services and to evaluate their possible effects on broader group of women of childbearing age who are potential users of such services [61,62]. A further exploration of this potential relationship would require both quantitative and qualitative research at the national and regional levels, examining the relationships between abortion policy and mental health in women from diverse cultural and socioeconomic backgrounds [63].

Strengths and limitations

The key strengths of the current study are its cross-national perspective, its reliance on the most recent and most reliable data available, and its consideration of the effects of abortion on women's mental health from a broader perspective. However, certain important limitations should be borne in mind when evaluating this study’s results. First, the data on access to legal abortion were based on legislation at the national level and cannot account for variations in access within the same country, such as differences across states [64]. Second, the estimated values for the prevalence of mental disorders in each country are based on a combination of data from the available literature and estimates based on statistical models; there may be a certain margin of error in these estimates, particularly in low- and middle-income countries [65]. Third, information on abortion policies provides only a partial picture of access to abortion - certain countries may have relatively “liberal” policies in theory, but women in these countries may encounter practical “informal” barriers to accessing abortion services [63,66]. Fourth, the findings of this study apply only at a broader population level and cannot be applied directly to individuals; it is not possible to conclude, based on these results, whether refusal of abortion would necessarily lead to depression or anxiety in a particular woman. Fifth, several other factors, such as religious beliefs, cultural values, social support, social welfare policies, and access to contraception, can potentially moderate the relationships that were observed in this study [67-70]. These were not examined due to the lack of reliable cross-national data on these variables, however, their inclusion in further statistical models is essential to obtain a more wide-ranging perspective on this subject. Finally, the age groupings provided by the Global Burden of Disease database (10-24 years and 25-49 years) are somewhat broad and artificial; the availability of more fine-grained data on the prevalence of common mental disorders in women of different age groups (e.g., ages 15-19, 19-24, 25-39 and 40-49 years) could have led to a more accurate analysis of the mediating role of age.

Recommendations

From a clinical and research perspective, it is hoped that the results of this study will lead to more rigorous analyses of the links between reproductive rights and mental health in women of childbearing age. This could include studies of attitudes and beliefs related to abortion policy in this population, and their association with factors, such as childhood adversity, social support, and cultural values, as well as longitudinal studies of mental health following abortion or abortion refusal in low- and middle-income countries.

From a policy perspective, these findings provide potential opportunities for collaboration between those involved on both “sides” of the abortion debate. Even if ideological differences preclude direct agreement on the issue of abortion, it may still be possible to find common ground on issues such as the protection of women from intimate partner violence, or on broader causes, such as gender development, gender equality, and women’s mental health, and to cooperate on policy changes relating to these causes.

## Conclusions

The results of the current study, though subject to certain limitations, suggest that there is a modest relationship between access to legal abortion and the mental health of women aged 25-49 years at the cross-national level. However, this relationship appears to be largely of an indirect nature and influenced by factors, such as gender development, gender inequality, and intimate partner violence. The issue of abortion has psychological and social implications that extend beyond the restricted group of women directly seeking abortion. Furthermore, this issue cannot be considered in isolation but must be examined in the context of broader social, cultural, and political factors that influence women’s rights, their autonomy, and their place in society.
